# Antidepressant Efficacy of Adjunctive Aerobic Activity and Associated Biomarkers in Major Depression: A 4-Week, Randomized, Single-Blind, Controlled Clinical Trial

**DOI:** 10.1371/journal.pone.0154195

**Published:** 2016-05-06

**Authors:** Cristiana Carvalho Siqueira, Leandro L. Valiengo, André F. Carvalho, Paulo Roberto Santos-Silva, Giovani Missio, Rafael T. de Sousa, Georgia Di Natale, Wagner F. Gattaz, Ricardo Alberto Moreno, Rodrigo Machado-Vieira

**Affiliations:** 1 Laboratory of Neuroscience LIM-27, Institute and Department of Psychiatry, University of Sao Paulo, Sao Paulo, Sao Paulo, Brazil; 2 Experimental Therapeutics and Pathophysiology Branch, National Institute of Mental Health (NIMH), Bethesda, Maryland, United States of America; 3 Laboratory of Movement Studies, University of Sao Paulo School of Medicine *Hospital das Clínicas*, Sao Paulo, Sao Paulo, Brazil; 4 Center for Interdisciplinary Research on Applied Neurosciences (NAPNA), University of Sao Paulo, Sao Paulo, Sao Paulo, Brazil; 5 Mood Disorders Unit (GRUDA), Institute and Department of Psychiatry, University of Sao Paulo, Sao Paulo, Sao Paulo, Brazil; 6 Department of Clinical Medicine and Translational Psychiatry Research Group, Faculty of Medicine, Federal University of Ceará, Fortaleza, CE, Brazil; Vanderbilt University, UNITED STATES

## Abstract

**Background:**

Major depressive disorder (MDD) is a highly prevalent, heterogeneous and systemic medical condition. Treatment options are limited, and recent studies have suggested that physical exercise can play an important role in the therapeutics of MDD. The aim of this study was to evaluate the antidepressant efficacy of adjunctive aerobic activity in association with pharmacotherapy (selective serotonin reuptake inhibitor) in symptomatic MDD as well as its association with physiological biomarkers.

**Methods:**

In this randomized, single-blind, add-on, controlled clinical trial, 57 patients (18–55 years of age) were followed-up for 28 days. All patients were drug-free, had been diagnosed with symptomatic MDD and received flexible dose of sertraline during the trial. Patients were randomized to either a 4-week program (4x/week) of add-on aerobic exercise (exercise group, N = 29) or no activity (control group, N = 28). Depression severity was assessed using the Hamilton Rating Scale for Depression (HAM-D) as the primary outcome. At baseline and endpoint, all patients underwent a comprehensive metabolic/cardiopulmonary exercise testing—including determination of maximal oxygen uptake (VO_2_max), VO_2_ at the second ventilatory threshold (VO_2_-VT2), and oxygen pulse (O_2_ pulse).

**Results:**

Depression scores significantly decreased in both groups after intervention. Importantly, patients in the aerobic exercise group required lower sertraline dose compared to the control group (sertraline monotherapy). The VO_2_max and O_2_ pulse parameters increased over time only in the exercise group and remained unchanged in the control group.

**Conclusions:**

The present findings suggest that a 4-week training of aerobic exercise significantly improves functional capacity in patients with MDD and may be associated with antidepressant efficacy. This approach may also decrease the need for higher doses of antidepressants to achieve response. Further studies in unmedicated and treatment-resistant MDD patients are needed in order to confirm the utility of short-term aerobic exercise as an alternative therapeutic approach in MDD.

**Trial Registration:**

ClinicalTrials.gov NCT02427789

## Introduction

Patients with Major Depressive Disorder (MDD) experience metabolic dysfunction associated with reduction in physical capacity and higher risk of death [1]. According to the World Health Organization [2], MDD is currently the fourth leading cause of disability worldwide and are projected to become the second leading cause by 2020.

Current treatments only improve partially or do not induce response in a significant percentage of patients. As alternative, non-pharmacological treatments for MDD have been studied and one important approach is physical exercise [3]. Initial studies have suggested that systematic physical exercise is associated with the prevention and improvement of depressive symptoms [4, 5]. Thus it represents a viable option for treatment as an add-on approach to pharmacotherapy. Physical exercise has a favorable side effect profile, low-cost and is widely accessible. It can be also tailored to the individual needs, depending on age, medical comorbidities, lifestyle and functional status [6, 7]. However, there is uncertainty regarding the physiological effects of physical exercise (e.g. duration and intensity necessary to bring about positive changes in mood and well-being) [3] underlying its potential antidepressant effects [8–13].

Studies have shown that exercise programs (4–12 weeks) are effective in reducing depressive symptoms [14] and at least two weeks are required to alter key neural cicuits in mood regulation [15, 16].

Physical exercise have been proposed as an alternative therapy to improve depression [8,11,15]. Cardiopulmonary exercise testing (CPET), which combines exercise testing and spirometry, is a key method to evaluate the physiological responses, ensuring greater safety and more accurate results [17]. This approach has never been applied systematically in clinical trials in depression. Previously, Sui et al. demonstrated an association between high cardiorespiratory fitness and a lower prevalence of depressive symptoms, independent of risk factors [18]. Similarly, Tolmunen et al. found that low maximal oxygen uptake (VO_2_max) were associated with greater severity of depressive symptoms in middle-aged men [19]. According to Blumenthal et al. [20], in order to produce improvement in depression, an exercise program should consist of high-intensity exercises (high energy expenditure). In contrast, Krogh et al. reported that the benefits of exercise in depression may be limited because its effects are seen only in the short-term [21]. In view of these inconclusive results regarding the effect of physical exercise on the symptoms of depression and the lack of concomitant evaluation of physiological biomarkers, this study investigated for the first time the clinical efficacy of short-term aerobic exercise in patients with MDD associated with its biological effects on a wide range of metabolic biomarkers.

## Materials and Methods

### Study design

This was a single-center study, conducted at the Institute of Psychiatry, University of São Paulo, Brazil. To be enrolled in this study, subjects had diagnosis of MDD assessed by the Mini International Neuropsychiatric Interview, in accordance with DSM-IV criteria [22]. All patients were drug-free at least 5 weeks before starting the study. Patients were randomly allocated to take part in a 4-week program of aerobic exercise (exercise group, n = 29) or not (control group, n = 28). Immediately randomization, all patients started with the selective serotonin reuptake inhibitor sertraline (50 mg/day), adjusted as necessary and not exceeding 100 mg/day. The primary outcome measure was the severity of depressive symptoms, as determined by the HAM-D scores. Secondary outcome measures were overall physical fitness and cardiac function and association with antidepressant response. Response was defined as a ≥ 50% reduction in the HAM-D score, and remission was defined as a HAM-D score < 8. All patients that dropped out during study was included in the statistical analysis by intention to treat Patients in the exercise group had 4 sessions/week for 28 days. The exercise intensity was pre-determined on the basis of the baseline cardiopulmonary function and HRs were monitored with the Borg scale [23]. To evaluate the dynamic response of ventilatory and metabolic variables to physical exercise, all patients underwent a CPET at baseline and endpoint, which included determination of VO2max, VO2 at the second ventilatory threshold (VO2-VT2), maximal heart rate (HRmax) and oxygen pulse (O2 pulse) as well as assessed with the Hamilton Rating Scale for Depression (HAM-D) and the Beck Depression Inventory (BDI). Exercise intensity (load) was initially set up at 60% of VO_2_max and progressively increased up to 85% of VO_2_max by the endoint. The exercise session consisted of continuous and intermittent aerobic activity; patients always had individualized and supervised sessions. All procedures were performed in accordance with the Declaration of Helsinki. The study was approved by the Commission of ethics for Analysis of Research Projects—CAPPesq (Protocol no. 8676, issued in July 19, 2012), and all subjects gave written informed consent.

### Subjects

In this randomized, single-blind, controlled clinical trial (ClinicalTrials.gov NCT02427789), 57 patients (ages 18 and 55 years) were recruited between November 2012 and August 2014 at our outpatient clinics (Mood Disorders Program, Laboratory of Neuroscience and Affective Disorders Group, GRUDA). At baseline, all patients scored at least 15 on the HAM-D rating scale (moderate to severe depression). To be included, patients should not have contraindication to exercise (any disabling medical condition); any cardiovascular disease, infection, neurological disorder, illicit drugs or alcohol abuse, medical comorbidity, active suicidal ideation and history of any DSM-IV Axis I psychiatric disorder. All patients included attended < 90% of the exercise sessions and weekly evaluations were performed by raters who were blinded to the group allocation. Patients in the control group were asked not to engage in any exercise during the study period.

**Fig 1:**
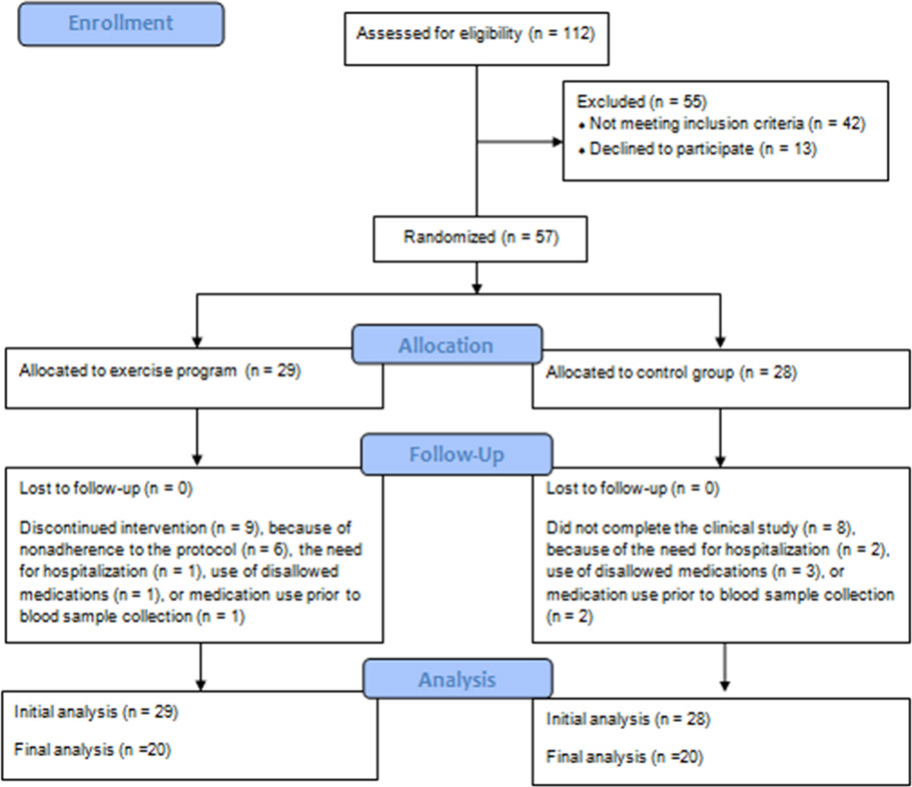
Flow chart of participants in the study.

We conducted 332 pre-screening telephone interviews, which yielded 112 individuals referred to the clinical screening (Fig 1). After the clinical screening, only 57 individuals were found to meet the study criteria. Of those 57 individuals, 40 (70.2%) completed the clinical study. The remaining 17 (29.8%) dropped out or were excluded for the following reasons: declining to continue in the study (n = 8), worsening of clinical symptoms and requiring hospitalization (n = 2), non-compliance to the exercise program (n = 11), use of other psychotropic medications (n = 4).

### Randomization and blinding

Randomization was done using randomly permuted blocks method (available from: www.randomization.com). Allocation was performed by a research assistant, who informed the researcher (RMV) the code for each patient. Patients were advised that information about the group to which they had been allocated should not be revealed to the rater. They were instructed to keep that information confidential until the end of their participation in the project.

### Sample size calculation

Sample size was estimated with G*Power software (available from the University of Dusseldorf: http://www.psycho.uni-dusseldorf.de/aap/projects/gpower/). To detect an effect size = 0.82, with a 5% risk of type I error, 20 patients per group were expected to be required. We also performed a sample size calculation, based on the study conducted by Schuch et al. [16], aimed at achieves a power of 80% at a 10% level of significance, resulting in an expected sample of 19 patients per group (two sample t-test). The expectation was that the reductions in the parameters evaluated would be 20% greater in the exercise group than in the control group.

### Statistical analysis

The normality and homogeneity of the data were tested and confirmed by the Kolmogorov-Smirnov and Shapiro-Wilk tests. For comparisons between the control group and exercise group, parametric Student’s t-tests for independent samples was used, whereas Student’s t-tests for paired samples was used to compare the pre- and post-intervention values. The primary outcome was analyzed using a mixed, repeated measures analysis of variance model with 1 dependent within-subject variable (HAM-D score), 1 within-subject variable (time, 2 levels), and 1 between-subject variable (group, 2 levels). Correlations between qualitative variables were evaluated with Fisher’s exact test or the chi-square test, whereas the correlations between quantitative variables were evaluated with Pearson’s correlation coefficient. Finally, regarding predictors of response, we performed multiple linear models (MANOVA) using the difference between baseline and endpoint scores as the dependent variable (HAM-D) and group and the putative predictor as the independent variables. The interaction was non-significant, thus we dropped the interaction terms and only used the main effects. The level of statistical significance was set at p < 0.05, and the data are presented as mean ± standard deviation (SD). The data were analyzed and processed with the SPSS Statistics software package, version 22.0 for Windows (IBM Corporation, Armonk, NY, USA).

## Results

### Participant characteristics

Forty-one women and 16 men (mean age 38.83 ±10.72 years) were enrolled. There were no significant differences between the groups regarding age (p = 0.507) or duration of illness (p = 0.84). Both groups had an above average education level (Table 1). Subjects in the exercise group used lower sertraline dose than controls. The correlation between the HAM-D scores and the dose of sertraline used by the exercise group patients, albeit not significant, showed a trend toward greater improvement, despite the use of lower doses of the antidepressant in comparison with the control group patients. (Table 1)

**Table 1:** Socio-demographic and medical characteristics in the exercise group and controls.

	Exercise (n = 29)	Control (n = 28)	*p*-value*
Age (years)- *mean (SD)*	39.76±11.60	37.86±9.85	0.506
Gender-n (%)			
- Female	21 (72.4%)	20 (71.0%)	0.9
- Male	8 (27.6%)	8 (29.0%)	
Education on Set- *n (%)*			
- Low	0 (0.0%)	6 (21.5%)	0.955
- Intermediate	11 (38.0%)	9 (32.1%)	
- High	18 (62.0%)	13 (46.4%)	
Smoker- *n (%)*			
- No	21 (72.4%)	26 (92.9%)	0.9
- Yes	8 (27.6%)	2 (7.1%)	
Professional status- *n (%)*			
- Employed	23 (79.4%)	24 (85.7%)	0.97
- Unemployed	1 (3.4%)	1 (3.6%)	
- Student	5 (17.2%)	3 (10.7%)	
Duration of illness- *mean (SD)*	53.89±64.16	51.64±64.12	0.846
Final dose of the antidepressant- **n (%)**			
- 50 mg	15 (52%)	5 (18%)	0.01
- 100 mg	5 (17%)	15 (54%)	
- without medicine	9 (31%)	8 (28%)	

Data are presented as n (%), mean (± SD), when appropriate.

*P-values by chi-squared test for categorical variables and by ANOVA for continuous variables.

**Fig 2:**
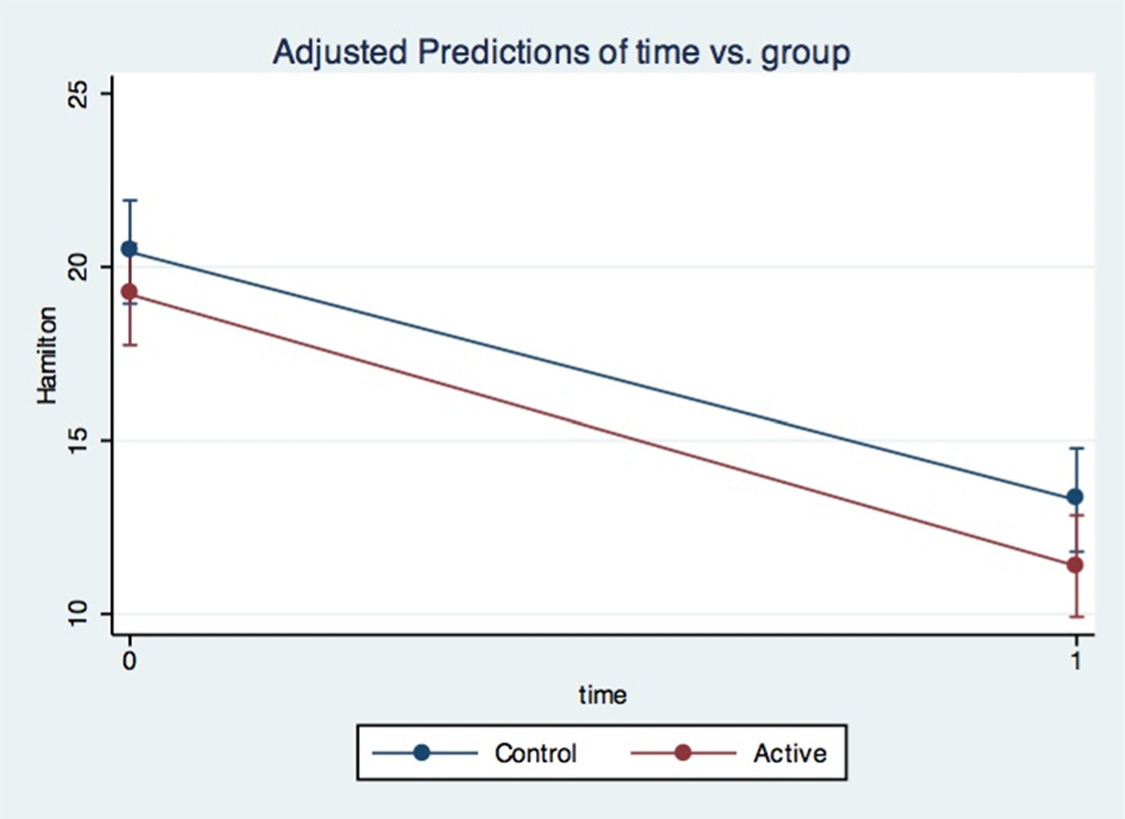
Hamilton Depression Rating Scale scores in the physical activity and control groups during a 4-week follow-up period.

We observed a significant main effect of time but not interaction between time and group in HAM-D (time F = 3.88; P = 0.05; *time X group* F = 1.99; P = 0.16). Similarly, regarding BDI scores, we observed a significant main effect for time but not the interaction time and group (time F = 50; P < .001; *time X group* F = 0.05; P = 0.82) (Fig 2).

**Fig 3:**
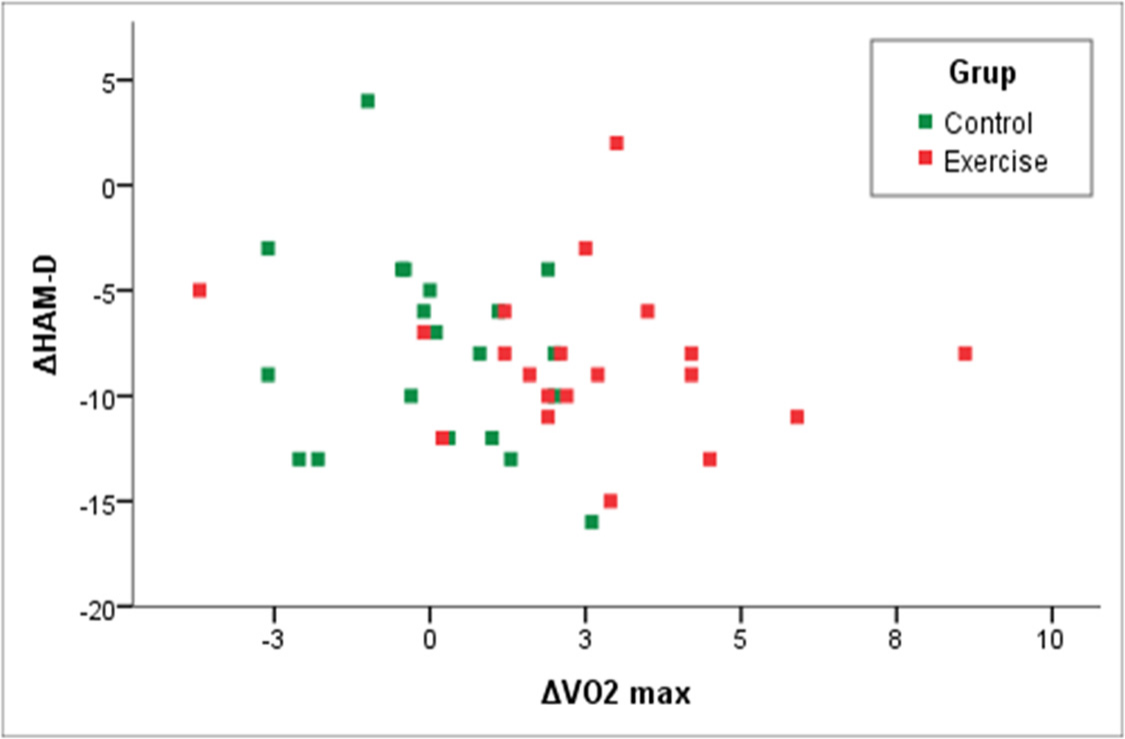
Correlation between changes in HAM-D score and VO_2_max from baseline to endpoint.

At the end of the 4-week trial, improvement in the HAM-D scores was similar in both groups (Fig 2). Physical activity did not show superiority compared to the control group regarding antidepressant improvement but improved VO2 max parameter (Fig 3).

At the endpoint, there was improvement in BDI scores in both groups. However, the changes in the HAM-D and BDI scores, even when considered both groups, were still not sufficiently significant to represent an association between a regular program of exercise and a reduction in the severity of depressive symptoms, control group (p = 0.15), or exercise group (p = 0.35) (Table 2).

**Table 2:** Pearson’s correlation coefficients for clinical scores and cardiopulmonary variables.

Variable	Changes in HAM-D score		Changes in BDI score	
	Exercise (p*)	Control (p*)	Exercise (p*)	Control (p*)
ΔVO_2_max	−0.58 (0.35)	−0.22 (0.15)	0.95 (0.19)	0.31 (0.83)
ΔHRmax	−0.02 (0.81)	−0.05 (0.79)	0.03 (0.87)	0.00 (0.50)
ΔO_2_ pulse	1.0 (0.46)	−0.01 (0.95)	1.22 (0.09)	0.03 (0.99)
ΔVO_2_-VT2	0.00 (0.67)	−0.02 (0.40)	0.00 (0.98)	−0.07 (0.751)

p*- value

As shown in Table 2, neither the changes in HAM-D or BDI scores were correlated with changes in VO_2_max, HRmax, O_2_ pulse, or VO2-VT2.

The VO_2_max (Table 3) remained stable over the study in the control group (p = 0.803) but significantly increased in the exercise group (p < 0.001). In the exercise group, O_2_ pulse also significantly elevated over the baseline value (p = 0.008), and the difference between the two groups, in terms of the final O_2_ pulse, was significant as well (p = 0.05). In addition, VO_2_-VT2 increased significantly in the exercise group (p = 0.01), although that was not the case in the control group (p = 0.297). Our exploratory analyses identified no variable as a potential predictor of response (Table 4).

**Table 3:** Comparison between baseline and final values for the study outcome measures by group.

Variable		Exercise^1^	Control^1^	Variation	Statistics^2^
HAM-D score	Baseline	19.20 ± 3.14	20.42 ± 2.99	-1.22	t = 1.50; p = 0.13
	Final	11.38 ± 3.94	12.64 ± 5.74	-1.26	t = 0.32; p = 0.16
	Statistics^2^	t = −8.36; p < 0.001	t = −6.38; **p < 0.001**		
BDI score	Baseline	29.51 ± 9.46	33.92 ± 9.64	-4.41	t = 1.74; p = 0.08
	Final	18.39 ± 9.00	20.05 ± 10.21	-1.66	t = 0.65; p = 0.51
	Statistics^2^	t = −4.58; p < 0.001	t = −5.22; **p < 0.001**		
VO_2_max score	Baseline	25.49 ± 5.95	26.31 ± 5.58	-1.35	t = 0.49; p = 0.49
	Final	27.57 ± 6.13	26.55 ± 5.46	1.17	t = -0.57; p = 0.53
	Statistics^2^	t = 4.577; p < 0.001	t = 0.253; p = 0.803		
O_2_ pulse score	Baseline	10.94 ± 2.38	10.43 ± 2.69	0.51	t = -0.68; p = 0.32
	Final	11.87 ± 2.57	10.47 ± 2.71	1.40	t = -1.71; **p = 0.05**
	Statistics^2^	t = 2.956; **p = 0.008**	t = 0.355; p = 0.727		
VO_2_-VT2 score	Baseline	20.24 ± 1.30	20.90 ± 0.80	-0.67	t = 0.43; p = 0.66
	Final	22.23 ± 1.09	21.60 ± 1.11	0.63	t = -0.40; p = 0.69
	Statistics^2^	t = 2.870; p = **0.010**	t = 1.073; p = 0.297		

^1^Results presented as means ± standard deviations.

^2^Student’s t-test for paired samples.

**Table 4:** Predictors of response in the total sample.

Predictor variable	Main effect		Group Effect		Interaction	
	F	p	F	p	F	p
Age (yrs)	0.06	0.81	2.08	0.15	2.19	0.14
Gender	0.01	0.93	0.22	0.63	0.61	0.44
ΔVO_2_max	1.42	0.24	0.00	0.99	0.29	0.59
ΔHRmax	0.13	0.71	0.24	0.62	0.02	0.89
ΔO_2_ pulse	0.63	0.43	0.18	0.67	0.58	0.44

## Discussion

This study aimed to evaluate the antidepressant effects of physical exercise adjunctive to pharmacotherapy (sertraline) and its influence on psychobiological aspects in patients with MDD. Depression severity significantly decreased in both groups but patients in the aerobic exercise group required lower sertraline dose compared to the control group. The VO_2_max and O_2_ pulse parameters selectively enhanced over time in the exercise group and were stable in the control group. By the end of the 4-week study period, we did not observe a specific association between a regular program of exercise and a reduction in the severity of depressive symptoms, as observed in previous studies [21,24,25]. The add-on exercise program improved cardiorespiratory fitness in adults; this program comprised 20–60 min of continuous or intermittent aerobic exercise and exercise intensity being set at 60–90% of HRmax or 50–85% of VO_2_max [26].

Despite the short duration of the exercise program, improvement in physical fitness and cardiac function was selectively observed in the exercise group. Specifically, improvement in cardiorespiratory fitness (increased VO_2_-VT2), aerobic capacity (increased VO_2_max), and oxygenation (increased O_2_ pulse) were observed. In addition, we observed that the exercise group used lower doses of antidepressant than controls and this correlation indicates a potential greater initial improvement in the exercise group.

The main strengths of the study were the use of a homogeneous sample and the Application of CPET for tailoring personalized exercise training. This distinguishing combines exercise testing and spirometry, and has not been done in previous comparable studies [15,16]. Different from previous studies in depression [3,11,12,27], we evaluated moderate to severe depression in an homogeneous sample of MDD patients and receiving monotherapy with sertraline. Therefore, the differences between our protocol and those described in the literature might explain the fact that we found exercise to have no influence on depressive symptoms. Potential limitations include the relative small sample size, the use of antidepressant (add-on design) and the short period of time. Another important point is that the HAM-D was not applied on a weekly basis, which could have allowed us to identify rapid antidepressant response.

There is no consensus regarding the role of exercise in treating depression. Our findings support a potential effectiveness as add-on therapy in MDD. As previously mentioned, Krogh et al. reported that exercise provides short-term benefits in patients with clinical depression [21]. In a recent study, Cooney et al. concluded that although exercise is more effective in treating depression than lack of treatment, exercise is not more effective than antidepressants or psychotherapy [28]. When those authors limited their analysis to high-quality studies, they found that the beneficial effects of exercise were significantly reduced.

## Conclusions

Although there is evidence supporting the role of exercise in reducing depressive symptoms, little is known of its clinical effectiveness due to methodological flaws, especially related to the exercise protocols, type of exercise (aerobic, anaerobic, or both) and intensity. Given potential biases and lack of evidence of the long-term efficacy of physical exercise in the treatment of depressive symptoms, we performed this protocol. Our findings demonstrated that aerobic exercise significantly improved several parameters related to cardiopulmonary capacity in MDD, which may underlie its antidepressant efficacy. This approach also decreased the need for higher doses of antidepressants to achieve antidepressant efficacy and thus represents a valuable adjunctive treatment during depressive episodes.

## Supporting Information

S1 File IRB approval. IRB final approval, original and English.
(PDF)

S2 File Consort Checklist. Consort checklist description.
(PDF)

S1 Protocol Registration of Research Protocol. Registration of Research Protocol, original and English.
(PDF)
