# Supplementary material for: Antidepressant Efficacy of Adjunctive Aerobic Activity and Associated Biomarkers in Major Depression: A 4-Week, Randomized, Single-Blind, Controlled Clinical Trial
Source: PLoS One. 2016 May 6;11(5):e0154195. doi: 10.1371/journal.pone.0154195 (PMC4859497; doi:10.1371/journal.pone.0154195)
Supplement: S1 File — IRB final approval, original and English. (PDF) [file pone.0154195.s001.pdf]

Hospital das Clínicas da FMUSP
Comissão de Ética para Análise de Projetos de Pesquisa - CAPPesq

PROJETO DE PESQUISA

Título: EFICÁCIA TERAPÊUTICA DO EXERCÍCIO FÍSICO ADJUNTIVO A FARMACOTERAPIA NA DEPRESSÃO MAIOR E ASSOCIAÇÃO COM BIOMARCADORES
Pesquisador Responsável: Prof. Dr. wagner Farid gattaz **Versão: 3**
Pesquisador Executante: Cristiana Carvalho Siqueira
Finalidade Acadêmica Mestrado
Instituição: HCFMUSP
Departamento: PSIQUIATRIA

PARECER CONSUBSTANCIADO DO CEP

Nº: 8676

Data da Relatoria: 18/07/2012

Apresentação do Projeto: Pesquisa de desenho claro para avaliar se paciente com transtorno depressivo maior em uso de tratamento medicamentoso associado ao exercício físico controlado como terapêutica adjuntiva na depressão, melhora a qualidade de vida, serão trabalhados 40 pacientes 20 em cada braço, sendo com exercício ou não

Objetivo da Pesquisa: Avaliar a eficácia clínica do exercício físico e sua influência nos aspectos psicobiológicos de pacientes deprimidos, através da avaliação dos níveis de preditores de resposta (biomarcadores), associados a depressão. Tais resultados contribuirão para a compreensão da fisiopatologia do TDM, fornecendo indícios para o diagnóstico, prognóstico, ou predição de resposta ao tratamento.

Avaliação dos Riscos e Benefícios: Adequado

Comentários e Considerações sobre a Pesquisa: Pesquisa adequada sem pendencia éticas

Considerações sobre os Termos de apresentação obrigatória: Adequado

Recomendações: Nenhuma

Conclusões ou Pendências e Lista de Inadequações: As solicitações foram atendidas

Situação do Parecer: Aprovado

Considerações Finais a critério do CEP: Aprovado

São Paulo, 19 de Julho de 2012

Prof. Dr. Luiz Eugênio Garcez Leme
Coordenador
Comissão de Ética para Análise de Projetos de Pesquisa-CAPPesq

Rua Dr. Ovídio Pires de Campos, 225 - Prédio da Administração - 5º andar
CEP 05403-010 - São Paulo - SP.
55 11 2661-6442 ramais: 16, 17, 18 e 20 | cappesq@hcnet.usp.br

Hospital das Clinicas, FMUSP

Ethics Committee for Research Project Analysis - CAPPesq

RESEARCH PROJECT

Title: THERAPEUTIC EFFECTIVENESS OF PHYSICAL EXERCISE ADJUNTIVE TO PHARMACOTERAPY IN MAJOR DEPRESSION AND ASSOCIATION WITH BIOMARKERS

Responsible researcher: Prof. Mr. Wagner Farid Gattaz **Version: 3**

Performer researcher: Cristiana Carvalho Siqueira

Academic Purpose: Master

Institution: HCFMUSP

Department: Psychiatry

CEP CONSUBSTANTIATED OPINION

No.: 8676

Date of rapporteurs: 18/07/2012

Project Presentation: Light design research to assess whether patients with major depressive disorder in use of drug treatment associated with the exercise controlled as adjunctive therapy in depression, improves quality of life, 40 patients will be worked out 20 in each arm, and with exercise or not.

Search goal: To evaluate the clinical efficacy of exercise and its influence on psychobiological aspects of depressed patients, by evaluating levels of response predictors (biomarkers) associated with depression. These results contribute to the understanding of the pathophysiology of MDD, providing evidence for the diagnosis, prognosis, or predicting response to treatment.

Risks and Benefits Rating: Suitable

Comments and Considerations for Search: without proper ethical disputes

Considerations Terms mandatory presentation: Suitable

Recommendations: No

Conclusions or To Do List and Inadequacies: The requests were met

Opinion of the situation: Approved

Concluding Remarks at the discretion of the CEP: Approved

São Paulo, July 19, 2012

Prof. Dr. Luiz Eugenio Garcez Leme

Coordinator

Ethics Committee for Research Project Analysis - CAPPesq

Dr. Ovidio Pires de Campos Street, 225 - Administration Building - 5th floor

CEP 05403-010 - São Paulo - SP

55 11 2661-6442 - extensions: 16, 17,18 and 20 / cappesq@hcnet.usp.br
