# Supplementary material for: Antidepressant Efficacy of Adjunctive Aerobic Activity and Associated Biomarkers in Major Depression: A 4-Week, Randomized, Single-Blind, Controlled Clinical Trial
Source: PLoS One. 2016 May 6;11(5):e0154195. doi: 10.1371/journal.pone.0154195 (PMC4859497; doi:10.1371/journal.pone.0154195)
Supplement: S1 Protocol — Registration of Research Protocol, original and English. (PDF) [file pone.0154195.s003.pdf]

PROTOCOLO DE ENTREGA DE NOVA PESQUISA
SECRETARIA DO CONSELHO DO DEPARTAMENTO DE PSIQUIATRIA DA FMUSP

DATA: 24 / 11 /2011

Nº Formuláro *on line:*	Título da Pesquisa	Pesquisador responsável	Pesquisador executante
8676	**EFICÁCIA TERAPÊUTICA DO EXERCÍCIO FÍSICO ADJUNTIVO A FARMACOTERAPIA NA DEPRESSÃO MAIOR E ASSOCIAÇÃO COM BIOMARCADORES**	**Prof. Dr. Wagner Farid Gattaz**	**Cristiana Carvalho Siqueira**

Pesquisador Principal: Prof.Dr. Wagner Farid Gattaz

Especialidade: Psiquiatria Grupo/Serviço : LIM27

Fone resid: (11) 30698010 celular: 11 98898887 fax: 11 3069 7535 email: gattaz@usp.br

Pesquisador Executante: Cristiana Carvalho Siqueira

Especialidade: Fisioterapia Grupo/Serviço : LIM 27 Externo:

Fone resid: 11 43057027 celular: 11 61654506 fax: email:cristianacsiqueira@ig.com.br

Informações obrigatórias:

- Pesquisa é de Iniciação a Pesquisa: Não
- Pesquisa de Pós-Graduação: Sim
- È pesquisador de Instituição externa: Não De qual:
- Tempo de finalização do projeto: 24 meses

Documentos apresentados:

- ☐ Pesquisa + CD
- ☐ Anexo I (tirado do site do CAPPesq)
- ☐ Formulário "*on line*"
- ☐ Parecer Consubstanciado
- ☐ Folha de rosto CONEP (apresentar somente quando solicitada pela Secretaria do ConDep)
- ☐ Carta de anuência (quando o projeto tiver parcerias com outros serviços ou instituições)
- ☐ Outros: **(expecificar)**

CLINICAL HOSPITAL THE MEDICAL SCHOOL OF THE UNIVERSITY OF SÃO PAULO

CLINIC BOARD

ETHICS COMMITTEE FOR ANALYSIS OF RESEARCH PROJECTS - CAPPesq

REGISTRATION OF RESEARCH PROTOCOL

Registration (be used by the Department of CAPPesq)

No. Protocol: Type: Human

Institute: IPQ

Online registration No: 8676 Date: 22/11/2011

This project involves:

Patients HC .... Yes

HC doctors or staff (as research subjects) .... No

Documents HC (Medical records and other) .... Yes

Materials stored in HC .... No

Anatomical parts of corpses .... No

1. Title of Research Protocol

THERAPEUTIC EFFECTIVENESS OF EXERCISE adjunctive pharmacotherapy IN GREATER DEPRESSION AND ASSOCIATION WITH BIOMARKERS

2. Key words characterizing the subject of search

major depressive disorder, exercise, interleukins, oxidative stress, BDNF, cortisol, spirometry, neuroprotection.

3. Summary of Research Protocol:

Depression is a disease that causes major harm on physical health and social life of the patient taking them to isolation, reducing their physical and professional ability and increasing the risk of death in these patients. It also knows the importance of exercise in maintaining quality of life and, most recently as adjunctive therapy in depression. The precise mechanisms by which exercise improves symptoms of depression are not yet very clear, but is likely to be multifactorial, including changes in the levels of monoamines, cortisol and neurotrophic factors. They are also known its effects on the improvement of cognitive functions, stimulating brain plasticity and resilience, as well as promoting self-esteem and social interaction. In this project we investigate additional findings that provide information about biomarkers involved in the pathophysiology of depression and treatment response to exercise. We expect to find by analyzing the levels of potential biomarkers associated with neuroprotective mechanisms (interleukins, markers of oxidative stress, BDNF, Cortisol), before and at the end of the proposed exercise program, response biomarkers. It will also assess whether the controlled exercise can be used as a therapeutic means to treat depression. To obtain the best expression of functional evaluation indices of the patient spirometry is used to test which will determine the variables respiratory, metabolic and cardiovascular that patient. So this project is based on the need for research adjunctive alternative treatments, more open and less conventional, which may form part of treatment strategies in depression.

4. Principal Investigator:

Prof. Dr. Wagner Farid Gattaz

http://lattes.cnpq.br/8681520193756072

Graduation: Physiotherapist

Link: HC

5. Researcher Performer:

Cristiana Carvalho Siqueira

http://lattes.cnpq.br/4917328956382490

6. you have co-authors?

Yes, Quantity: 1

Names of co-authors: Rodrigo Machado Vieira

7. Where the search will be carried out?

Department: Psychiatry

Discipline: Psychiatry

LIM: LIM / 27 - Neuroscience Lab

8. There is attributable to external agencies?

No

9. It has foreign participation

No

10. The project is multicenter

No

11. Other Services / HCFMUSP divisions involved in research

Yes

Institute of Orthopedics

12. Purpose of academic research and classification

Master's degree

13. Research

Prospective

14. Materials and methods

Laboratory

Interviews and questionnaires

Records of patients

15. Gender, Search classification

Trial

16. Thematic areas set out in Res. 196/96

New Procedures

19. Search Execution Schedule

Term: 24 months

20. Subscriptions

Signature and stamp Researcher Approved _____ / ______ / _______.

Signature and stamp of the Head

dated approval

by the Department Council approved in _____ / ______ / _______.
